# Increases in [^3^H]Muscimol and [^3^H]Flumazenil Binding in the Dorsolateral Prefrontal Cortex in Schizophrenia Are Linked to α4 and γ2S mRNA Levels Respectively

**DOI:** 10.1371/journal.pone.0052724

**Published:** 2013-01-08

**Authors:** Mathieu Verdurand, Stu G. Fillman, Cynthia Shannon Weickert, Katerina Zavitsanou

**Affiliations:** 1 Schizophrenia Research Institute, Sydney, Australia; 2 Schizophrenia Research Laboratory, Neuroscience Research Australia, Sydney, Australia; 3 School of Psychiatry, University of New South Wales, Sydney, Australia; 4 ANSTO LifeSciences, Australian Nuclear Science and Technology Organization, Sydney, Australia; McLean Hospital/Harvard Medical School, United States of America

## Abstract

**Background:**

GABA_A_ receptors (GABA_A_R) are composed of several subunits that determine sensitivity to drugs, synaptic localisation and function. Recent studies suggest that agonists targeting selective GABA_A_R subunits may have therapeutic value against the cognitive impairments observed in schizophrenia. In this study, we determined whether GABA_A_R binding deficits exist in the dorsolateral prefrontal cortex (DLPFC) of people with schizophrenia and tested if changes in GABA_A_R binding are related to the changes in subunit mRNAs. The GABA orthosteric and the benzodiazepine allosteric binding sites were assessed autoradiographically using [^3^H]Muscimol and [^3^H]Flumazenil, respectively, in a large cohort of individuals with schizophrenia (n = 37) and their matched controls (n = 37). We measured, using qPCR, mRNA of β (β1, β2, β3), γ (γ1, γ2, γ2S for short and γ2L for long isoform, γ3) and δ subunits and used our previous measurements of GABA_A_R α subunit mRNAs in order to relate mRNAs and binding through correlation and regression analysis.

**Results:**

Significant increases in both [^3^H]Muscimol (p = 0.016) and [^3^H]Flumazenil (p = 0.012) binding were found in the DLPFC of schizophrenia patients. Expression levels of mRNA subunits measured did not show any significant difference in schizophrenia compared to controls. Regression analysis revealed that in schizophrenia, the [^3^H]Muscimol binding variance was most related to α4 mRNA levels and the [^3^H]Flumazenil binding variance was most related to γ2S subunit mRNA levels. [^3^H]Muscimol and [^3^H]Flumazenil binding were not affected by the lifetime anti-psychotics dose (chlorpromazine equivalent).

**Conclusions:**

We report parallel increases in orthosteric and allosteric GABA_A_R binding sites in the DLPFC in schizophrenia that may be related to a “shift” in subunit composition towards α4 and γ2S respectively, which may compromise normal GABAergic modulation and function. Our results may have implications for the development of treatment strategies that target specific GABA_A_R receptor subunits.

## Introduction

The majority of inhibition in the cerebral cortex of the mammalian brain is mediated by the interaction of the neurotransmitter γ-aminobutyric acid (GABA) with ionotropic GABA_A_ receptors (GABA_A_R). These ligand-gated channels allow chloride ions to enter neurons, hyperpolarising their membrane and attenuating their activity. Dysfunctional cortical inhibition has been suggested as a mechanism through which symptoms of schizophrenia are mediated [1].

The functional GABA_A_R is composed of five subunits, typically two α's, two β's and a single γ/δ or ε subunit, assembled into a pentamer. Nineteen subunits of the GABA_A_R can be grouped together in 8 families through sequence homology (α1–6, β1–3, γ1–3, δ, ε, θ, π, ρ1–3) [2]. The prevalent γ2 subunit has a well-known splice variant in which exon 10 (24 base pairs) is removed producing the γ2 short isoform (γ2S) [3], [4]. Multiple binding sites are found on the GABA_A_R with an orthosteric binding site for GABA at the interface of the α and β subunits and an allosteric binding site for benzodiazepines at the junction of an α (α1, α2, α3 or α5) and a γ subunit [2], [5]–[9]. The specific subunits present determine the pharmacological sensitivity, synaptic localisation and function of the GABA_A_R [10] and these subunits change during normal human cortical development as well as in disease states [5], [11]–[14].

Changes in the expression and function of GABA_A_R have been strongly implicated in schizophrenia. Binding studies targeting the orthosteric site of GABA_A_R have robustly showed an increase in [^3^H]Muscimol binding particularly in the dorsolateral prefrontal cortex (DLPFC), and also in the caudate nucleus, posterior and anterior cingulate cortices, superior temporal gyrus, and hippocampal formation of post-mortem schizophrenic brains compared to controls [15]–[22]. This finding has been reinforced by the observation of increased GABA_A_R protein subunit α2 in the prefrontal cortex in schizophrenia [23]. However, studies on benzodiazepine binding (to the allosteric GABA_A_ receptor site) in schizophrenia show conflicting results: Increased [^3^H]Flunitrazepam binding has been reported across a variety of cortical areas including the medial frontal cortex, orbitofrontal cortex, temporal gyrus, and the parahippocampal cortex with the DLPFC yet to be assessed [24], [25]. Other post-mortem [26] or imaging [27]–[29] studies have reported no changes or decreased [30] benzodiazepine binding in schizophrenia.

At the mRNA level, results have also been inconsistent with studies reporting an increase in α1 and α5 [31], a decrease in α1, α5 and β2 [12], a decrease in γ2S [32] and our own group observing no diagnostic change in α1–4 and a decrease in α5 in people with schizophrenia [11]. Presumably, a substantial heterogeneity in the pathophysiology of schizophrenia coupled with small cohort sizes contributes to the lack of consistency amongst the above findings. Clarification on the alterations in the expression of GABA_A_R subunits is important as they may contribute to changes in both the orthosteric and allosteric binding sites in schizophrenia [5], [33], [34] affecting pharmacological receptor properties and response to treatment. However, these parameters have not been studied together in the same cohort.

Therefore, in the present study, we used a fairly large cohort of 37 schizophrenia cases and their matched controls to examine whether deficits in the GABA orthosteric and benzodiazepine allosteric binding site of the GABA_A_R exist in the DLPFC in schizophrenia. An important aspect of our study was to determine, in the same cohort, if mRNA expression of the subunits involved in the formation of these binding sites (α and β for GABA binding site and α and γ or δ for benzodiazepine binding site) were also different in schizophrenia and to determine how they may relate to changes in binding levels.

## Materials and Methods

### 2.1. Human post-mortem brain samples and ethics statement

All research was conducted in accordance with the latest version of the declaration of Helsinki and approved by the Human Research Ethics Committees at the University of Wollongong (#HE99/22) and at the University of New South Wales (#HREC0761) that follow the guidelines set out in the National Statement on Ethical Conduct in Research involving humans (http:/www.nhmrc.gov.au). Written consent for use of tissues in the study was obtained from next of kin. Characterization and tissue preparation for this Australian schizophrenia cohort has been described in detail previously [35]. Tissue samples and frozen cryostat sections were prepared from the large cohort of schizophrenia (n = 37) and controls (n = 37) cases matched for age, gender, pH, and post-mortem interval (see Table 1 for demographics information) [35].

**Table 1:** Summary of Cohort Demographics.

	n	Age at death (yrs)	pH	PMI (hrs)	Freezer Storage (Months)	Brain Volume (ml)	Brain Weight (g)	RIN	Age of Onset	Duration of Illness	Lifetime chlorpromazine (mg)
Control	37	51.1±14.6	6.7±0.3	24.8±11.0	69.6±42.7	1438.2±121.7	1446.4±127.1	7.3±0.6	*NA*	*NA*	*NA*
Schizophrenia	37	51.3±14.1	6.6±0.3	28.5±13.8	79.9±37.2	1408.1±165.5	1394.3±164.0	7.3±0.6	23.7±6.1	27.6±13.8	7.9±8.0×10^6^
Depressive§	4	52.3±24.0	6.9±0.1	27.5±15.8	46.8±20.7	1517.3±125.3	1490.0±94.2	7.4±0.5	24.5±9.3	27.8±21.3	4.4±5.5×10^6^
Bipolar§	3	50.7±14.6	6.6±0.3	23.7±5.9	71.7±29.7	1449.7±95.7	1396.7±105.0	7.2±0.3	29.3±2.1	21.3±12.5	1.1±1.0×10^6^
Paranoid	16	51.6±13.0	6.6±0.3	32.2±16.0	83.6±27.5	1379.4±184.1	1364.4±177.7	7.2±0.6	23.6±6.4	28.1±12.8	7.7±7.8×10^6^
Disorganized	5	54.0±24.2	6.7±0.3	25.7±6.8	74.8±42.8	1406.6±190.5	1385.2±184.2	7.2±0.7	19.8±1.8	34.2±12.5	8.9±4.3×10^6^
Undifferentiated	7	48.6±16.9	6.5±0.2	28.0±14.9	82.0±53.2	1420.3±147.0	1433.0±175.1	8.0±0.5	21.9±3.9	27.0±16.4	9.9±1.2×10^6^
Residual	2	51.0±0.0	6.1±0.6	16.5±6.4	136.0±4.2	1317.0±231.9	1325.0±233.3	6.6±0.5	31.0±5.7	20.0±5.7	3.4±1.8×10^6^

Abbreviations: yrs, years; hrs, hours; PMI, post-mortem interval; RIN, RNA integrity number.

Average values ± SD for continuous variables are shown.

§ Schizoaffective.

### 2.2. Tissue dissection and section preparation

**Figure 1:**
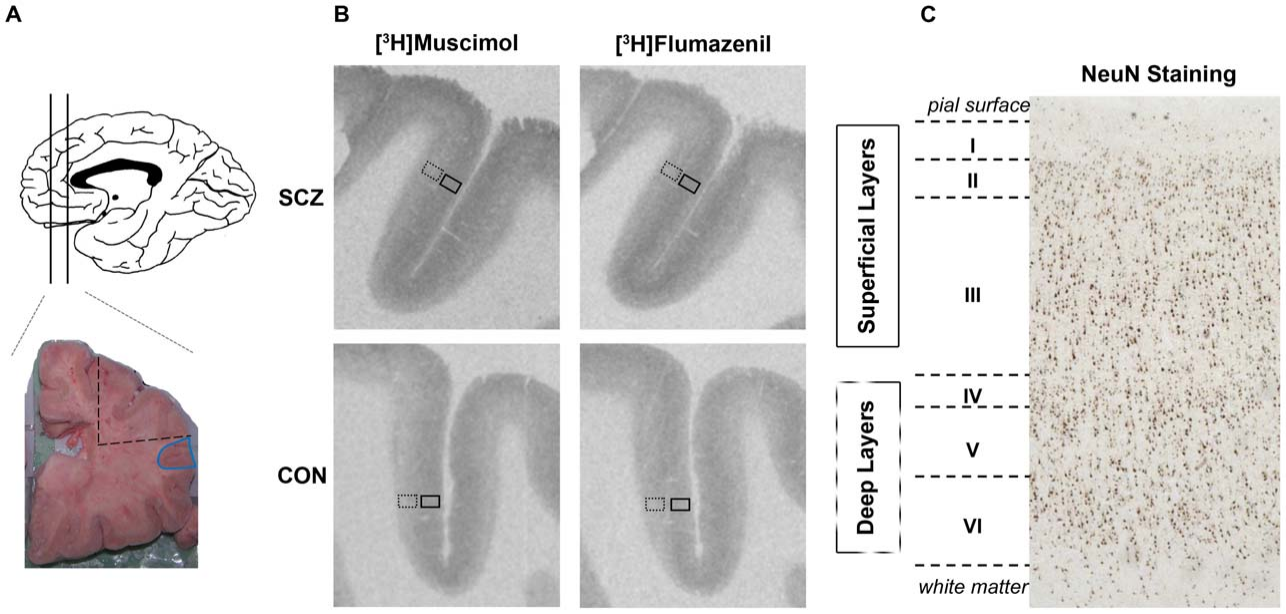
An overview of the experimental procedures used in the autoradiography experiments. A Sagittal view of brain with lines indicating area of coronal section with the blue outlined area is where homogenate tissue was isolated with the dashed lines indicating the bounds of tissue slices used for autoradiography (A). Typical autoradiographs (B) obtained with [^3^H]Muscimol (GABA orthosteric binding site agonist) and [^3^H]Flumazenil (benzodiazepine allosteric binding site antagonist) in the DLPFC of control (CON) and schizophrenia (SCZ) cases. NeuN staining (C) that allowed identification of the cortical layers in the DLPFC is presented on the right side of the figure, along with the drawing methodology used to quantify binding in the “superficial” (layers I, II, and III) and “deep” layers (layers IV, V and VI) of the DLPFC. The superficial layers quantification “box” is indicated in solid line, while the deep layers quantification “box” is indicated in dotted line.

Tissue dissection has been described in detail previously [35]. Briefly, at autopsy, brain weight and volume were determined [36]. The fresh tissue was cut into ∼1 cm coronal slices and various anatomical areas were dissected for separate freezing. For the DLPFC (Brodmann's area 46, Figure 1A) dissections, frozen tissue was dissected on a dry ice platform using a dental drill (Cat# UP500-UG33, Brasseler, USA) for homogenates. DLPFC tissue (average weight of tissue ∼0.5 g grey matter tissue from the crown of the middle frontal gyrus) was obtained from an adjacent coronal slab corresponding to the middle one-third (rostral caudally) found anterior to the genu of the corpus callosum. Coronal tissue sections of the DLPFC containing the superior and inferior frontal sulcus were cut (14 µm) on a cryostat, thaw mounted onto microscope slides and stored at −80°C until use.

### 2.3. In vitro autoradiography

[^3^H]Muscimol autoradiography was carried out based on the method described previously [17], [21], [30] with minor modifications. All sections were processed simultaneously to minimize experimental variance. On the day of the experiment, sections were pre-incubated three times for 5 min at 4°C in 50 mM Tris citrate buffer (pH 7.0). Sections were then incubated for 60 min at RT in the same buffer with the addition of 20 nM [^3^H]Muscimol (specific activity 35.6 Ci/mmol, Perkin Elmer, USA). Non-specific binding was determined by incubating adjacent sections in the same solution with the addition of 100 µM GABA (Sigma). The concentration of [^3^H]Muscimol was measured in 10 µl aliquots taken from the incubation mixture. After the incubation, sections were washed two times for 1 minute in cold (4°C) 50 mM Tris citrate buffer (pH 7.0). Sections were then dipped briefly in cold distilled water and then air dried.

[^3^H]Flumazenil (Ro-15-1788) autoradiography was carried out based on the method described in Hand et al., 1997 [37] with minor modifications. On the day of the experiment, sections were pre-incubated for 20 min at room temperature in 50 mM Tris (pH 7.4). Sections were then incubated for 80 min at room temperature in the same buffer with the addition of 5 nM [^3^H]Flumazenil (specific activity 83.4 Ci/mmol, Perkin Elmer, USA). Non-specific binding was determined by incubating adjacent sections in the same solution but in the presence of 10 µM flumazenil (Sigma). The concentration of [^3^H]Flumazenil was measured in 10 µl aliquots taken from the incubation mixture. After the incubation, sections were washed two times for 1 minute in cold 50 mM Tris buffer (pH 7.4). Sections were then dipped briefly in cold distilled water and then dried.

Following the assays, dried sections were apposed to Kodak Biomax MR films together with autoradiographic standards ([^3^H]microscales from Amersham), in x-ray film cassettes. Films were developed after 8 weeks ([^3^H]Muscimol) and 4 weeks ([^3^H]Flumazenil) using Kodak GBX developer and fixed with Kodak GBX fixer.

### 2.4. Quantitative analysis of autoradiographic images

All films were analyzed by using a computer-assisted image analysis system, Multi-Analyst, connected to a GS-690 Imaging Densitometer (Bio-Rad, USA). Quantification of receptor binding in DLPFC was performed by measuring the average optical density in adjacent brain sections. Both ligands presented a similar distribution binding pattern across the grey matter of the DLPFC with “superficial layers” (I, II and III) having higher binding compared to “deep layers” (IV, V and VI) (Figure 1B). Non-specific binding was found to be negligible (<5%) for both [^3^H]Muscimol and [^3^H]Flumazenil binding. Optical density measurements for specific binding were then converted into fmoles of radioligand per mg of tissue equivalent (fmol/mg TE), according to the calibration curve obtained from the [^3^H]microscales standards.

### 2.5. Total RNA isolation and RNA quality assessment

Total RNA was extracted from ∼300 mg of frozen tissue per subject using Trizol (Invitrogen, Carlsbad, California) according to the manufacturer's instructions (Kozlovsky et al., 2004). The quality of extracted total RNA was determined using the Agilent Bioanalyzer 2100 (Agilent Technologies, Palo Alto, California). A volume containing 100–200 ng RNA was applied to an RNA 6000 Nano LabChip, without heating before loading. The RNA integrity number (RIN) was used as an indicator of RNA quality, ranging from 1 (lowest quality) to 10 (highest quality). The cDNA was synthesized in three reactions of 3 µg of total RNA in a 26.25 µl reaction using the Superscript First-Strand Synthesis Kit (Invitrogen) according to the manufacturer's protocol.

### 2.6. Quantitative real-time PCR (qPCR)

GABA_A_R subunits mRNA levels were measured using a TaqMan Gene Expression Assays (Applied Biosystems) for GABRB1 (Hs00181306_m1), GABRB2 (Hs00241451_m1), GABRB3 (Hs00241459_m1), GABRG1 (Hs00381554_m1), GABRG2 (Long and Short isoforms, Forward Primer: 5′-CCAAGCAAGGACAAAGATAAAAAGAA-3′, Reverse Primer: 5′-TGCTGATCTTGGGCGGATAT-3′, Probe Short: AAAACCCTGCCCCTACC, Probe Long: ATGTTTTCCTTCAAGGCC, Figure S1), and GABRG3 (Hs00264276_m1). Each 10 µl qPCR reaction contained FAM-labeled probe (250 nmol/l), primers (900 noml/l), and 1.14 ng cDNA in 1× TaqMan Universal Mastermix containing AmpliTaq Gold DNA polymerase, deoxynucleoside triphosphates, uracil-N-glycosylase, and passive reference. The PCR protocol used involved incubation at 50°C for 2 min and 95°C for 10 min, followed by 40 consecutive cycles of 95°C for 15 sec and 60°C for 1 min. Serial dilutions of pooled cDNA (combined from all cases) were included on every qPCR plate and used by Sequence Detection Software (SDS; Applied Biosystems) to quantify sample expression through the relative standard curve method. Control wells containing no cDNA template displayed no amplification in any assay. Efficiencies of the qPCR reactions ranged from 77 to 100%, with r^2^ values of between 0.95 and 1.00. All reactions were performed in triplicate. Expression levels were normalized to the geometric mean of four “housekeeper” genes that did not change expression with diagnosis (all genes t's(>69)<0.86, p's>0.4): ACTB (Hs99999903_m1), GAPDH (Hs99999905_m1), UBC (Hs00824723_m1), and TBP (Hs00427620_m1) [35], [38]. Calculations of housekeeper stability were performed using geNorm (medgen.ugent.be/genorm) with resulting M-values of 0.15, 0.19, 0.25 and 0.30 for ACTB, GAPDH, UBC, and TBP respectively. For further detail on “housekeeper” gene selection, variance and stability please refer to Weickert et al. 2010 [35].

### 2.7. Statistical analysis

Statistical analyses were conducted using PASW Statistics 18.0 (SPSS, IBM) and GraphPad Prism (CA, USA) statistical packages. The data were normally distributed. All cases were included in the analyses. Mean values for binding and mRNA expression are reported ± standard error mean (SEM). Student's *t*-tests were used to compare the mean brain pH, age at death, PMI, freezer storage time, brain weight, brain volume and RIN between the schizophrenia and control groups. We tested the continuous variables (brain pH, age at death, PMI, freezer storage time, brain weight, brain volume, RIN, age of illness onset, illness duration, and estimated lifetime exposure to antipsychotics) for significant Pearson's correlations with binding and mRNA subunits expression. Non-continuous variables such as gender (male/female), hemisphere (right/left), cause of death (suicide/other), daily alcohol intake (none 0, low: 1, moderate: 2, and high: 3), and tobacco smoking (moderate: 1, and heavy: 2) were used as grouping variables with *t*-tests or one-way ANOVA to evaluate their effects on binding and mRNA expression.

GABA_A_R bindings between diagnostic groups (schizophrenia and control) were analysed using two-way analysis of covariance (ANCOVA), with diagnosis (schizophrenia and control) and layers (superficial and deep) as independent variables and co-varying for continuous variables that were significantly correlated with binding. GABA_A_R subunit mRNA expression levels between diagnostic groups (schizophrenia and control) were analysed using ANCOVA, co-varying for continuous variables that were significantly correlated with mRNA subunit expression.

To assess the relative implication of GABA_A_R mRNA subunits on binding measures in patients with schizophrenia and controls, we used forward regression analysis with all α and β mRNA subunits as predictors of the GABA orthosteric binding site targeted with [^3^H]Muscimol, and all α, γ and δ mRNA subunits as predicators of the benzodiazepine allosteric binding site, in both layers of the DLPFC. The criterion probability of F for predicators to enter the regression model was set at ≤0.05.

## Results

The mean age, pH, PMI, freezer storage time, brain weight, brain volume and RIN did not differ between the schizophrenia and control groups as shown by non-significant Student *t*-tests (age at death: t(72) = 0.057, p = 0.955; pH: t(72) = −0.644, p = 0.521; PMI: t(72) = 1.264, p = 0.21; freezer storage time t(72) = 1.102, p = 0.274; brain weight t(72) = −1.525, p = 0.132; brain volume t(72) = −0.893, p = 0.375; and RIN t(72) = −0.243, p = 0.809).

### 3.1. Disease related effects on [^3^H]Muscimol and [^3^H]Flumazenil binding

**Figure 2:**
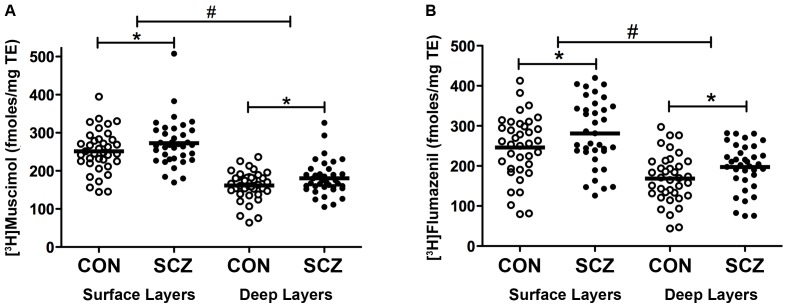
Scatter plots presenting the binding values (fmoles/mg tissue equivalent) of [^3^H]Muscimol (A) and [^3^H]Flumazenil (B) in control (CON) (n = 37) and schizophrenia (SCZ) (n = 37), in the superficial (surface) and deep layers of the DLPFC. * = p<0.05 and refers to a significant main effect of diagnosis (schizophrenia>control); # = p<0.001 and refers to a significant main effect of layers (superficial>deep) after two-way ANCOVA. Bars represent the means.

Significant associations were found between [^3^H]Muscimol binding and age and freezer storage time in both superficial and deep layers (age: −0.523<r<−0.524, p<0.001, freezer storage: 0.403<r<0.425, p<0.001, see Table S1 for details and section 3.4). In the whole cohort, these associations accounted for less than 20% of the variance and were co-varied for by performing two-way ANCOVA. This analysis revealed [^3^H]Muscimol binding varied with diagnosis (mean ± SEM: superficial layers control = 251.5±9.1 vs. schizophrenia = 272.9±10.0; deep layers control = 161.5±6.3 vs. schizophrenia = 180.4±7.5 fmol/mg TE, F(1,142) = 5.98, p = 0.016). [^3^H]Muscimol binding also varied with layer (F(1,142) = 184.5, p<0.001) with no interaction between the variables (F(1,142) = 0.033, p = 0.885). We found higher levels of [^3^H]Muscimol binding in DLPFC of people with schizophrenia compared to controls and the variation with layer was due to higher levels of [^3^H]Muscimol binding in superficial layers of the DLPFC compared to deep layers (see Figure 2).

For [^3^H]Flumazenil binding, significant associations were observed between and continuous variables in both superficial and deep layers (pH: 0.289<r<0.411, p<0.01, freezer storage: 0.434<r<0.526, p<0.001, see Table S1 for details and section 3.4), that accounted for less than 28% of the variance and were co-varied for in the ANCOVA. [^3^H]Flumazenil binding (mean ± SEM: superficial layers control = 246.2±13.0 vs. schizophrenia = 280.8±14.0; deep layers control = 168.5±10.1 vs. schizophrenia = 197.2±9.7 fmol/mg TE) varied with diagnosis (F(1,140) = 6.5, p = 0.012) and with layers (F(1,140) = 70.5, p<0.001) but no significant interaction between diagnosis and layers was found (F(1,140) = 0.095, p = 0.758). [^3^H]Flumazenil binding was higher in schizophrenia compared to controls and higher in the superficial layers of the DLPFC compared to deep layers (see Figure 2).

In schizophrenia, [^3^H]Muscimol and [^3^H]Flumazenil binding were significantly correlated in both the superficial (r = 0.434, p = 0.008) and deep layers (r = 0.399, p = 0.016). In the control group, these correlations approached significance (r = 0.299, p = 0.073 in superficial and r = 0.310, p = 0.062 in deep layers).

### 3.2. Disease related effects on GABA_A_R mRNA subunits expression (βs and γs)

**Figure 3:**
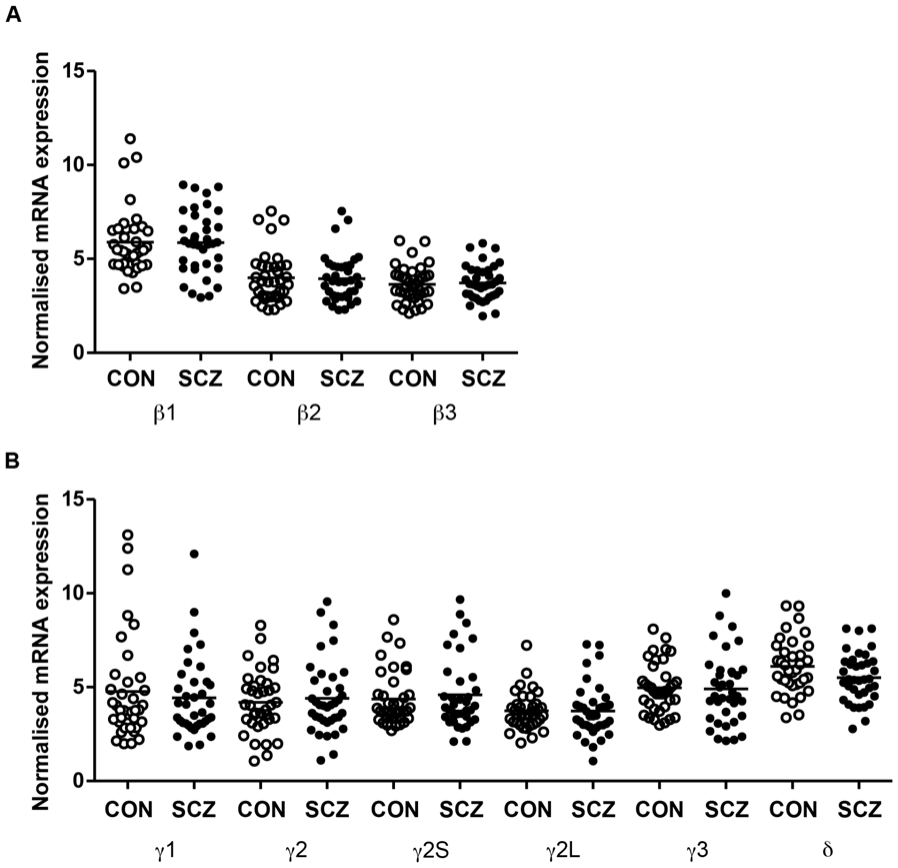
Scatter plots of the normalised expression level of GABA_A_R mRNA subunits (qRT-PCR) in the DLPFC of control (CON) (n = 37) and schizophrenia (SCZ) (n = 37) cases. (A) β mRNA subunits and (B) γ and δ mRNA subunits. Bars represent the means.

One-way ANCOVA (controlling for demographic variables significantly correlated to GABA_A_R mRNA subunit expression) revealed no significant effect of diagnosis (Figure 3) in any of the nine transcripts examined (β1–β3, γ1, γ2, γ2S, γ2L, γ3 and δ) (all F's<3.4, all p's>0.05).

### 3.3. Correlation and regression analysis

Pearson's correlation between [^3^H]Muscimol binding and α (measured in our previous study [11]) and β mRNA subunits are presented in Table 2 with separate analyses in the whole cohort, in patients with schizophrenia only, and in controls only. Pearson's correlation between [^3^H]Flumazenil binding and α, γ and δ mRNA subunits are presented in Table 3 with separate analyses in the whole cohort, in patients with schizophrenia only, and in controls only.

**Table 2:** Correlations between [^3^H]Muscimol binding and α-β GABA_A_R subunit mRNAs.

mRNA		All individuals *(n = 74)*		Schizophrenia *(n = 37)*		Control *(n = 37)*	
		Superficial Layers	Deep Layers	Superficial Layers	Deep Layers	Superficial Layers	Deep Layers
α1	*r*	***0.380***	***0.323***	***0.455***	***0.381***	0.306	0.273
	*p*	***0.001***	***0.005***	***0.005***	***0.020***	0.065	0.102
α2	*r*	***0.239***	0.213	***0.342***	0.310	0.140	0.117
	*p*	***0.043***	0.072	***0.041***	0.066	0.415	0.498
α3	*r*	0.191	***0.239***	0.236	0.295	0.119	0.148
	*p*	0.106	***0.041***	0.160	0.077	0.490	0.390
α4	*r*	***0.396***	***0.354***	***0.582***	***0.474***	0.172	0.211
	*p*	***0.001***	***0.002***	***<0.001***	***0.004***	0.309	0.210
α5	*r*	0.085	0.130	0.199	0.252	0.078	0.144
	*p*	0.472	0.269	0.238	0.133	0.646	0.395
β1	*r*	0.076	0.139	0.164	0.224	−0.013	0.056
	*p*	0.522	0.236	0.331	0.183	0.941	0.742
β2	*r*	***0.327***	***0.372***	***0.384***	***0.387***	0.249	***0.376***
	*p*	***0.004***	***0.001***	***0.019***	***0.018***	0.137	***0.022***
β3	*r*	***0.343***	***0.306***	***0.377***	0.304	0.306	0.310
	*p*	***0.003***	***0.008***	***0.021***	0.068	0.066	0.062

**Table 3:** Correlations between [^3^H]Flumazenil binding and α-γ/δ GABA_A_R subunit mRNAs.

mRNA		All individuals *(n = 74)*		Schizophrenia *(n = 37)*		Control *(n = 37)*	
		Superficial Layers	Deep Layers	Superficial Layers	Deep Layers	Superficial Layers	Deep Layers
α1	*r*	***0.382***	***0.300***	***0.390***	0.175	***0.406***	***0.475***
	*p*	***0.001***	***0.010***	***0.019***	0.309	***0.013***	***0.003***
α2	*r*	*−0.233*	−0.227	−0.025	−0.079	*−0.447*	*−0.372*
	*p*	*0.050*	0.057	0.888	0.653	*0.006*	*0.025*
α3	*r*	0.111	0.140	0.113	0.061	0.107	0.248
	*p*	0.352	0.240	0.510	0.722	0.534	0.144
α4	*r*	0.210	0.160	***0.333***	0.156	0.078	0.178
	*p*	0.076	0.180	***0.050***	0.370	0.644	0.291
α5	*r*	−0.038	−0.029	0.056	0.016	−0.017	0.054
	*p*	0.753	0.807	0.746	0.925	0.921	0.750
γ1	*r*	*−0.347*	*−0.270*	−0.199	−0.062	*−0.462*	*−0.407*
	*p*	*0.003*	*0.021*	0.245	0.718	*0.004*	*0.013*
γ2	*r*	−0.103	−0.149	0.058	−0.038	*−0.337*	−0.314
	*p*	0.387	0.210	0.736	0.824	*0.041*	0.058
γ2S	*r*	***0.441***	***0.393***	***0.553***	***0.364***	0.280	***0.426***
	*p*	***<0.001***	***0.001***	***<0.001***	***0.029***	0.094	***0.009***
γ2L	*r*	***0.270***	***0.248***	***0.352***	0.287	0.167	0.223
	*p*	***0.021***	***0.034***	***0.035***	0.089	0.322	0.185
γ3	*r*	0.046	0.069	0.268	0.298	−0.274	−0.222
	*p*	0.699	0.559	0.114	0.077	0.101	0.186
δ	*r*	−0.114	−0.140	0.012	0.032	−0.155	−0.204
	*p*	0.337	0.237	0.942	0.855	0.359	0.227

**Figure 4:**
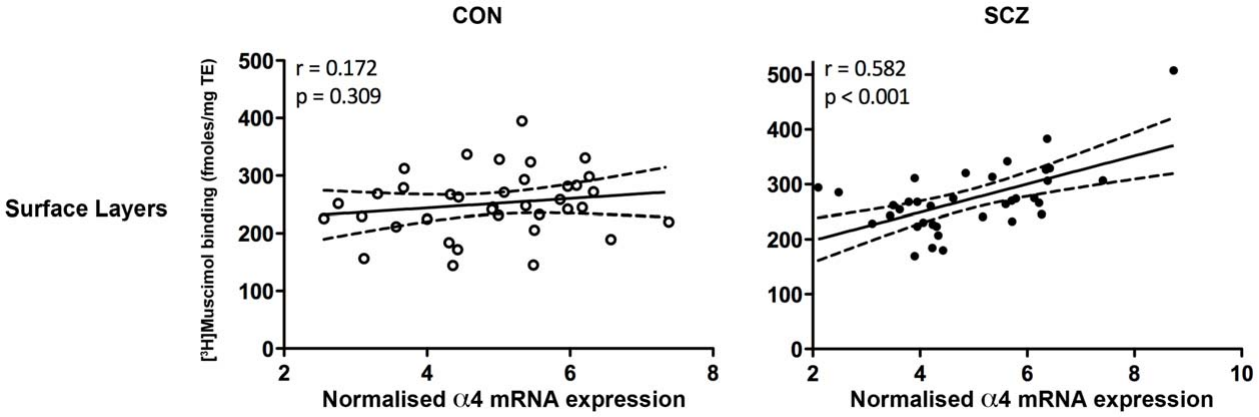
Correlations of [^3^H]Muscimol binding and α4 mRNA subunit expression level in the superficial layers of the DLPFC of control (CON) (n = 37) and schizophrenia (SCZ) (n = 37) cases.

In patients with schizophrenia, we used forward regression analysis with all α and β mRNA subunits (constituting the GABA orthosteric binding site) as predictors for [^3^H]Muscimol binding in both layers of the DLPFC. Interestingly, the α4 subunit mRNA was most strongly associated with binding and alone could account for 34% and 23% of the variance in [^3^H]Muscimol binding in superficial and deep layers of the DLPFC respectively (see Figure 4 for correlation plot and Table 4 for regression results). In contrast, in the superficial layers of the control group, no predictors (mRNA subunits) passed the criterion probability of F to enter the regression model. In the deep layers however, β2 mRNA subunit contributed the most (14%) to [^3^H]Muscimol variance in controls (Table 4).

**Table 4:** Regression modelling of the interaction between relevant GABA_A_R subunits (α and β) and [^3^H]Muscimol binding.

Group	Area	Method	Model	Variable (F-to-enter≤0.05)	*B*	*Std Error B*	*β*	*p value*	*R^2^*	*ΔR^2^*
Schizophrenia *(n = 37)*	Superficial Layers	Forward (α1,2,3,4,5,β1,2,3)	1	Constant	143.21	32.29		<0.001	.34	.34
				α4 mRNA	26.19	6.30	.59	<0.001	.34	.34
	Deep Layers	Forward (α1,2,3,4,5,β1,2,3)	1	Constant	101.74	25.97		<0.001	.23	.23
				α4 mRNA	15.83	5.07	.48	0.004	.23	.23
Control *(n = 37)*	Superficial Layers	Forward (α1,2,3,4,5,β1,2,3)		*none with F*≤*0.050*	*na*	*na*	*na*	*na*	*na*	*na*
	Deep Layers	Forward (α1,2,3,4,5,β1,2,3)	1	Constant	88.45	31.04		0.007	.14	.14
				β2 mRNA	18.31	7.65	.38	0.022	.14	.14

**Figure 5:**
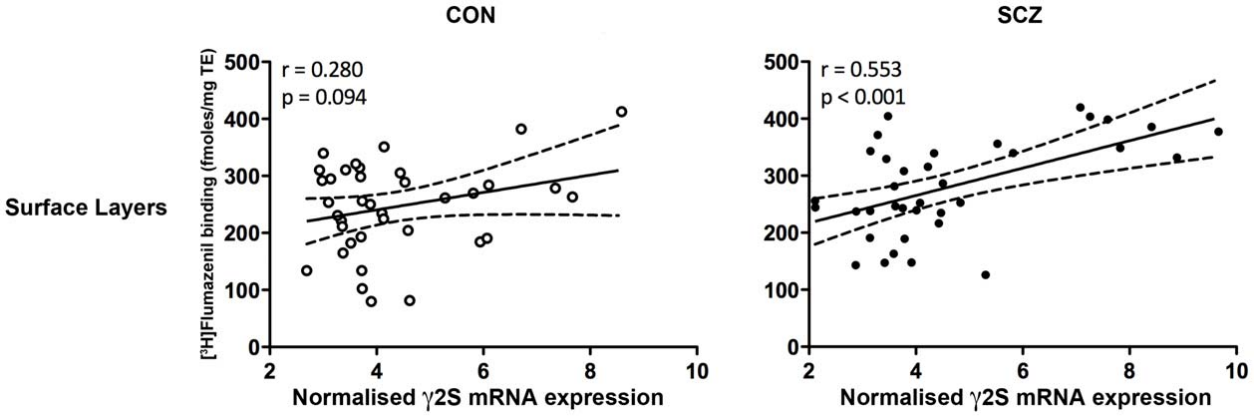
Correlations of [^3^H]Flumazenil binding and γ2S mRNA subunit expression level in the superficial layers of the DLPFC of control (CON) (n = 37) and schizophrenia (SCZ) (n = 37) cases.

In patients with schizophrenia, we used forward regression analysis with all α, γ and δ mRNA subunits (constituting the benzodiazepine allosteric binding site) as predictors of [^3^H]Flumazenil binding in both layers of the DLPFC. Interestingly, the γ2S subunit mRNA was most strongly associated with binding and alone could account for 31% and 13% of the variance in [^3^H]Flumazenil binding in superficial and deep layers of the DLPFC respectively (see Figure 5 for correlation plot and Table 5 for regression results). In contrast, when applying the same regression method in the control group, several mRNA subunits contributed to [^3^H]Flumazenil variance, including γ1, γ2 pan and γ2L in the superficial layers and α1, α2, γ2 pan and γ2S in the deep layers of the DLPFC (Table 5).

**Table 5:** Regression modelling of the interaction between relevant GABA_A_R subunits (α, γ and δ) and [^3^H]Flumazenil binding.

Group	Area	Method	Model	Variable (F-to-enter≤0.05)	*B*	*Std Error B*	*β*	*p value*	*R^2^*	*ΔR^2^*
Schizophrenia *(n = 37)*	Superficial Layers	Forward (α1,2,3,4,5,γ1,2,3,γ2L,γ2S,δ)	1	Constant	170.12	31.39		<0.001	.31	.31
				γ2 mRNA Short	24.08	6.31	.553	0.001	.31	.31
	Deep Layers	Forward (α1,2,3,4,5,γ1,2,3,γ2L,γ2S,δ)	1	Constant	146.85	24.28		<0.001	.13	.13
				γ2 mRNA Short	10.95	4.88	.364	0.032	.13	.13
Control *(n = 37)*	Superficial Layers	Forward (α1,2,3,4,5,γ1,2,3,γ2L,γ2S,δ)	1	Constant	308.69	23.75		<0.001	.21	.21
				γ1 mRNA	−13.10	4.31	−.462	0.005	.21	.21
			2	Constant	396.66	37.31		<0.001	.37	.16
				γ1 mRNA	−14.53	3.94	−.512	0.001	.37	.16
				γ2 mRNA Pan	−19.33	6.70	−.401	0.007	.37	.16
			3	Constant	318.27	44.96		<0.001	.49	.12
				γ1 mRNA	−15.70	3.64	−.554	<0.001	.49	.12
				γ2 mRNA Pan	−24.57	6.44	−.510	0.001	.49	.12
				γ2 mRNA Long	28.29	10.53	.358	0.011	.49	.12
	Deep Layers	Forward (α1,2,3,4,5,γ1,2,3,γ2L,γ2S,δ)	1	Constant	69.77	32.66		0.040	.23	.23
				α1 mRNA	21.95	6.97	.475	0.003	.23	.23
			2	Constant	118.63	38.59		0.004	.32	.09
				α1 mRNA	21.71	6.64	.470	0.003	.32	.09
				γ2 mRNA Pan	−11.39	5.34	−.307	0.040	.32	.09
			3	Constant	218.49	52.13		<0.001	.44	.12
				α1 mRNA	18.22	6.26	.394	0.006	.44	.12
				γ2 mRNA Pan	−13.94	5.01	−.375	0.009	.44	.12
				α2 mRNA	−14.84	5.66	−.361	0.013	.44	.12
			4	Constant	187.56	49.93		0.001	.53	.09
				α1 mRNA	12.77	6.20	.276	0.048	.53	.09
				γ2 mRNA Pan	−12.86	4.67	−.346	0.010	.53	.09
				α2 mRNA	−16.81	5.31	−.409	0.003	.53	.09
				γ2 mRNA Short	13.88	5.57	.330	0.018	.53	.09

### 3.4. Effect of continuous and non-continuous variables

In the whole cohort and in each diagnostic group, age at death was negatively correlated with [^3^H]Muscimol binding in both layers of the DLPFC (all r<−0.3, all p<0.05), freezer storage was positively correlated with [^3^H]Flumazenil in both layers (all r>0.3, all p<0.04) and with [^3^H]Muscimol in deep layers (all r>0.35, all p<0.02), and pH was positively correlated with [^3^H]Flumazenil in superficial layers only (all r>0.4, all p<0.01). Other significant correlations between GABA_A_R binding and continuous variables were found and are presented in Table S1, along with significant correlations between GABA_A_R mRNA subunit expression and continuous variables.

The *t*-tests for gender showed no significant male/female variation in binding measures (data not shown). Concerning mRNA subunits, only α4 showed a significant increase in males compared to females (+15.2%, t(71) = 2.003, p = 0.049). All the other subunits did not show a statistically significant change according to gender (data not shown).

The hemisphere analysed, right or left, did not have any significant effect on binding or mRNA subunits measures (data not shown). Cases who died by suicide in the schizophrenia group were characterised by an increase in [^3^H]Muscimol binding in the superficial (+19.5%, t(35) = 2.2, p = 0.035) and in the deep (+23.9%, t(35) = 2.4, p = 0.021) layers of the DLPFC compared to cases who died by natural means, whereas [^3^H]Flumazenil binding did not differ according to manner of death (superficial: t(34) = 0.2, p = 0.824; deep: t(34) = 0.1, p = 0.932). Concerning the mRNA subunits expression, all α1, α4 and α5 were significantly increased in the DLPFC of schizophrenia cases who committed suicide compared to the ones who did not (α1:+37.3%, t(35) = 2.6, p = 0.013), α4: +23.9%, t(34) = 2.1, p = 0.047 α5: +29.5%, t(35) = 2.6, p = 0.013).

In the schizophrenia group the amount of daily alcohol intake (none 0, low: 1, moderate: 2, and high: 3) resulted in greater [^3^H]Flumazenil binding in the deep layers of cases with high alcohol intake compared to those with no alcohol intake (ANOVA: F(3,29) = 2.871, p = 0.053, post-hoc: high alcohol and no alcohol, p = 0.018), but had no significant effect on [^3^H]Muscimol measures or mRNA subunit expression. The tobacco smoking habits during life of the individuals with schizophrenia (moderate: 0, and heavy: 1) had no significant impact on either the binding measures or mRNA subunit expression, with the exception of γ1 subunit that was higher in heavy smokers compared to moderate smokers (t(21) = 2.729, p = 0.013).

The lifetime anti-psychotic dose (chlorpromazine equivalent) did not have any significant impact on either binding measures (r's: −0.3<r<−0.2 and all p's>0.07) or mRNA subunits expression (r's: −0.3<r<0.1, all p's>0.08). The age of onset of schizophrenia did not have any significant effect on either binding measures (r's: −0.3<r<0.1 and all p's>0.08) or mRNA subunits expression (r's: −0.3<r<0.1, all p's>0.07). The duration of illness (in years) was negatively correlated with [^3^H]Muscimol binding measures in the superficial (r = −0.632, p<0.001) and deep layers (r = −0.584, p's<0.001) of schizophrenia patients but not with [^3^H]Flumazenil binding (r's: −0.3<r<−0.1, and all p's>0.1). The mRNA subunits expression for α1 (r = −0.374, p = 0.023) and α4 (r = −0.462, p = 0.005) were negatively correlated with duration of illness whereas the other subunits were not (r's: −0.3<r<0.2, all p>0.07).

## Discussion

### 4.1. Disease-related effects

We report increases of both the GABA and benzodiazepine binding sites of the GABA_A_R in the DLPFC in schizophrenia that are linked to α4 and γ2S mRNA subunits respectively. Our findings are in line with studies reporting increases in [^3^H]Muscimol binding (GABA binding site) in the DLPFC [18] and other cortical regions [16], [21]. The increase we observed in the benzodiazepine binding site in the DLPFC in schizophrenia confirms previous studies which report increases across a variety of cortical areas including the medial frontal cortex, orbitofrontal cortex, temporal gyrus, and the parahippocampal cortex [24], [25] but contradicts other studies that found no changes [26] or decreased binding in individuals with schizophrenia [30].

It is known that many demographic and peri-mortem factors including medication before death can influence binding and/or mRNA expression in post-mortem studies. Although in the present study several associations between these factors and binding/mRNA expression were identified, the sizes of these associations in the whole cohort were small and taken into account in the ANCOVA analysis. Moreover, no significant correlations between [^3^H]Muscimol and [^3^H]Flumazenil binding and antipsychotic medication (lifetime chlorpromazine) were found in the schizophrenia group. This suggests that the observed increases in GABA_A_R binding are not secondary to medication or demographic and/or peri-mortem factors.

We found significant correlations between [^3^H]Muscimol and [^3^H]Flumazenil binding in the whole cohort and in the schizophrenia group, but not in the control group. While the association between [^3^H]Flumazenil and [^3^H]Muscimol binding was statistically significant only in the patient group it cannot be discounted that a similar, though weaker, association was present in controls. This suggests that in general there is a positive association between the orthosteric GABA and allosteric benzodiazepine binding site but this is stronger in the disease state. The increases in [^3^H]Muscimol and [^3^H]Flumazenil binding we observed in the present study were not accompanied by cohort-wide changes in any of GABA_A_R subunit mRNA measured here. Despite this lack of overall change in primary transcript levels in schizophrenia, regression analysis revealed that, in the schizophrenia group, the α4 subunit mRNA levels contributed the most to the [^3^H]Muscimol binding variance in both layers of the DLPFC. In contrast in the control group, no mRNA reached a significant level of correlation with binding to enter the regression model in the superficial layers, whereas in the deep layers, β2 mRNA was the one mainly contributing to [^3^H]Muscimol binding variance.

The γ2 subunit is an important functional determinant of GABA_A_ receptors and is essential for formation of high-affinity benzodiazepine binding sites [39]–[41]. Using transgenic mice, Baer et al (2000) demonstrated that expression of either the long or the short γ2 splice variant resulted in mice that had indistinguishable [^3^H]Flumazenil binding, γ2 protein levels and phenotypes [42]. It is interesting that mRNA levels encoding the γ2S subunit was the strongest predictor of [^3^H]Flumazenil variance in both cortical layers of people with schizophrenia but also predicted a relatively small proportion of [^3^H]Flumazenil binding variance in deep cortical layers of controls. This implies that γ2S may be contributing more to binding in the disease state which would suggest that functional coupling, downstream of receptor activation, using PKC may be diminished in schizophrenia regardless of increased binding. In contrast, in the control group, several mRNA subunits were contributing to the variance in [^3^H]Flumazenil binding in the superficial and deep layers of the DLPFC. These observations suggest that the relationships between binding and mRNA expression at the benzodiazepine binding site are probably indirect and quite varied amongst the control population.

Overall the results of our regression analysis suggests that there may be a “shift” in the subunit composition of GABA_A_R towards inclusion of α4 when studying the GABA orthosteric binding site and γ2S when analysing the benzodiazepine allosteric binding site in schizophrenia.

### 4.2. Functional and therapeutic implications

Our results have several functional and therapeutic implications. The increases in both [^3^H]Muscimol and [^3^H]Flumazenil binding together with the putative “shift” in the subunit composition support the theory of altered GABAergic neurotransmission in the DLPFC. The increases in binding observed in the present study and others [18], [20], [23], [27], [28] could be due to compensatory adaptations for reduced GABA synthesis [11], [43]–[47] and reuptake [48] at parvalbumin-positive chandelier neurons that synapse on the axon initial segment of pyramidal cells [26], [53], [54]. However, compensation at GABA_A_R level has been suggested to be insufficient to restore the synchronized oscillatory activity of cortical pyramidal neurons, in the gamma band range, necessary for normal cognitive functioning [49]. If a dysfunctional GABA system in schizophrenia does lead to cognitive impairments, therapeutic strategies to correct or modulate disrupted GABAergic pathways could be used to treat cognitive symptoms of schizophrenia, regardless of whether these GABAergic deficits are primary or compensatory [50].

Our results point to therapeutic potential of drugs that target α4 or γ2S subunits in the treatment of symptoms of schizophrenia. GABA_A_R that include α4 subunits are generally insensitive to diazepam, the prototypical benzodiazepine [51]. If, as we propose, schizophrenia patients undergo a “shift” in their subunit composition of GABA_A_R towards α4 subunits, this could account for the 50% failure of response to benzodiazepine prescribed as “add-on” therapy in schizophrenia [52], [53]. Moreover, GABA_A_R that include α4 subunits, assembled with a γ or δ subunit [50], are located at extrasynaptic sites and are responsible for tonic inhibition. Tonic inhibition is defined as the constant activation of extrasynaptic receptors that reduces the probability of generating an action potential [54]. A “shift” in the subunit composition of GABA_A_R towards α4 subunits in our schizophrenia cohort might therefore reflect an abnormal shift towards extrasynaptic GABA_A_R mediated tonic inhibition, that could compromise normal GABAergic modulation of cortical excitability, contributing to schizophrenia symptoms.

GABA_A_R containing the γ2 subunit predominantly mediate phasic inhibition and have a synaptic localisation [50]. Phasic inhibition is defined as the rapid and synchronous opening of synaptic receptors that results in an inhibitory postsynaptic potential [54]. The long isoform of γ2 subunit (γ2L) has been shown to preferentially accumulate at synapses through the protein kinase C (PKC) phosphorylation of Ser343. Conversely, the γ2S isoform lacking Ser343 [7], [8] might not be able to “transfer” to synapses and rather localise extrasynaptically. In our schizophrenia cohort, a putative “shift” in the subunit composition of GABA_A_R towards γ2S isoforms possibly reflects a similar “shift” in the ratio of extrasynaptic/synaptic GABA_A_R as we suggested for α4 subunits. Alternatively, an increase in the γ2S isoform may be linked to down-stream changes in PKC phosphorylation signalling associated with abnormal modulation and function of the GABA_A_R in schizophrenia.

Therefore, increased contributions of both α4 and γ2S subunits to the formation of the orthosteric and allosteric GABA_A_R binding sites in schizophrenia, may result in an increase of GABA_A_Rs located extrasynaptically, and thus affect tonic inhibition of cortical neurons. Although the implication of synaptic GABA_A_R (controlling phasic inhibition) in the generation of rhythmic activity in neuronal networks (thus cognitive processes) is established [54], the role of extrasynaptic GABA_A_R (responsible for tonic inhibition) is still unclear. Mutant mice with a loss of tonic inhibition in the hippocampus exhibited an increase in the power of gamma band oscillations [55] and increased gamma band power at baseline (non-evoked) has been reported for people with schizophrenia [56]. It is possible that a dysfunction in tonic inhibition affects gamma band oscillations resulting in cognitive impairments in schizophrenia. Interestingly, neurosteroids acting on extrasynaptic GABA_A_R have been found to improve cognition in schizophrenia patients, suggesting that extrasynaptic receptors could be involved in cognitive impairments in schizophrenia [57], [58]. These studies, together with ours, reinforce the interest in developing therapeutics that target extrasynaptic GABA_A_Rs for the treatment of cognitive symptoms in schizophrenia.

## Conclusions

Our study showed parallel up-regulation of the GABA orthosteric and benzodiazepine allosteric GABA_A_R binding sites in the DLPFC of a large cohort of schizophrenia patients and controls. We reported associations between α4/γ2S mRNA subunit and binding that may suggest a shift in GABA_A_R subunit composition in schizophrenia affecting receptor localization and function and may have implications for the development of novel treatment strategies.

## Supporting Information

Figure S1 **Design for the custom GABA_A_R γ2 long and short variants TaqMan assay.** Probes (in grey) span exon-exon junctions in order to eliminate genomic signals. One set of primers was used to amplify transcripts both including and excluding exon 10 (in green).
(TIF)

Table S1 **Pearson's correlations between continuous variables, binding, and mRNA expression.**
(XLS)
