# Supplementary figures and images for: Increases in [3H]Muscimol and [3H]Flumazenil Binding in the Dorsolateral Prefrontal Cortex in Schizophrenia Are Linked to α4 and γ2S mRNA Levels Respectively
Source: PLoS One. 2013 Jan 8;8(1):e52724. doi: 10.1371/journal.pone.0052724 (PMC3540049; doi:10.1371/journal.pone.0052724)

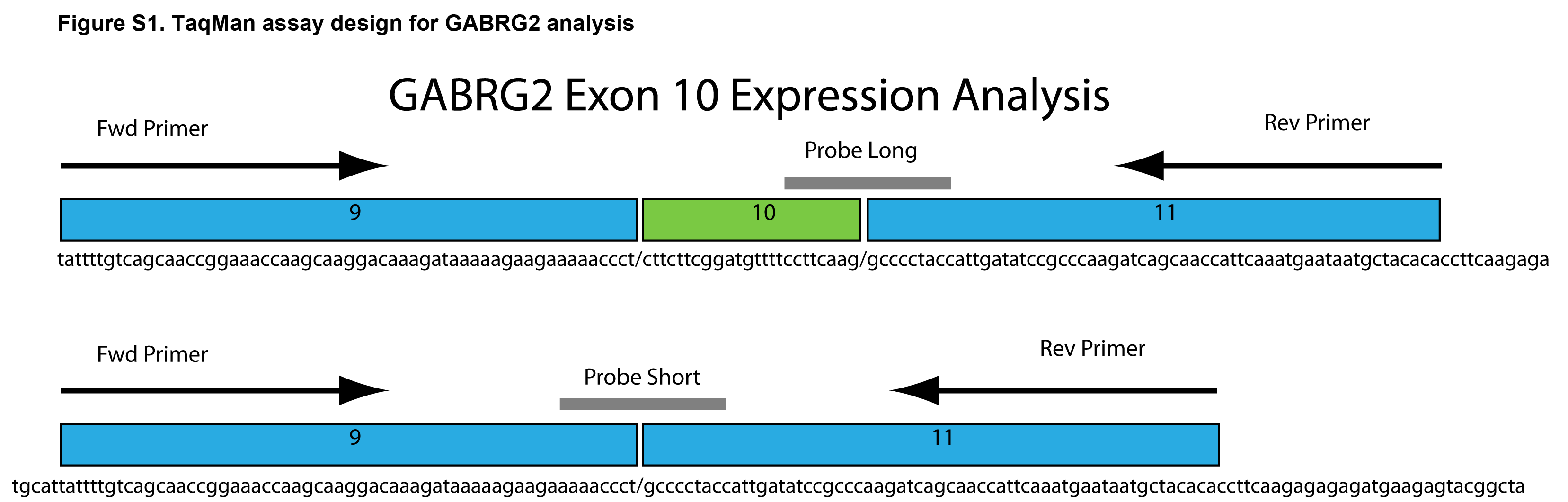

Supplement: Figure S1 — Design for the custom GABAAR γ2 long and short variants TaqMan assay. Probes (in grey) span exon-exon junctions in order to eliminate genomic signals. One set of primers was used to amplify transcripts both including and excluding exon 10 (in green). (TIF) [file pone.0052724.s001.tif]
